# Supplementary material for: Lactuca super-pangenome reduces bias towards reference genes in lettuce research
Source: BMC Plant Biol. 2024 Oct 28;24:1019. doi: 10.1186/s12870-024-05712-2 (PMC11514843; doi:10.1186/s12870-024-05712-2)
Supplement: Supplementary file 2 — Supplementary Material 2: Supplementary figure S1) Functional enrichment of core Lactuca genes. Supplementary figure S2) Functional enrichment of variable Lactuca genes. Supplementary figure S3) CNV in the RLL2B gene in L. sativa. Supplementary figure S4) Completeness assessment for both v8 and v11 of L. sativa var. Salinas proteome. [file 12870_2024_5712_MOESM2_ESM.pdf]

Supplementary Figures

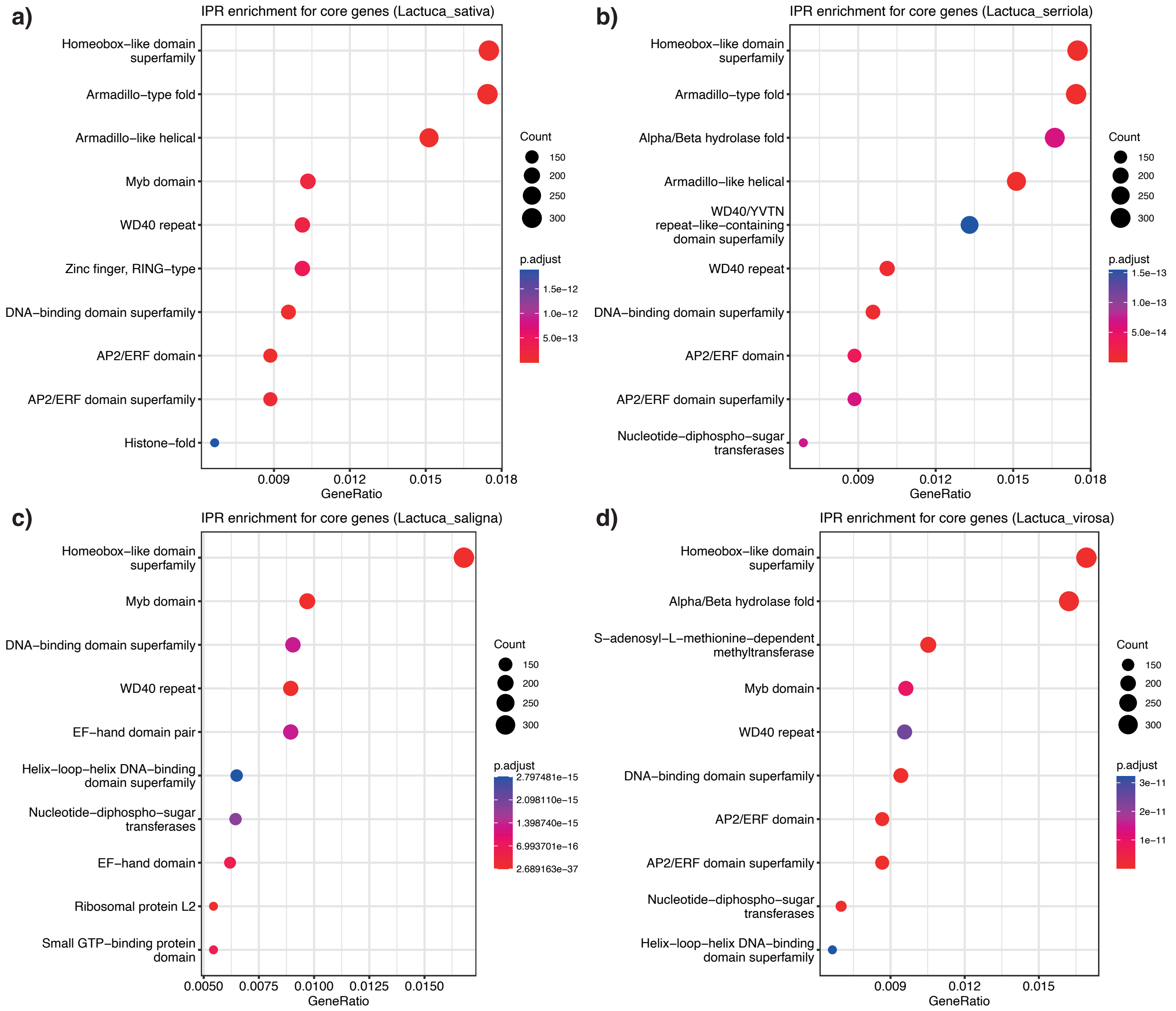

Supplementary Figure S1: Functional enrichment of the core *Lactuca* genes in *Lactuca sativa* (**a**), *Lactuca serriola* (**b**), *Lactuca saligna* (**c**) and *Lactuca virosa* (**d**) using InterPro domains. Only the first ten most significant domains are shown (p-value adjusted according to Bonferroni). For full results, see Supplementary Data 8.

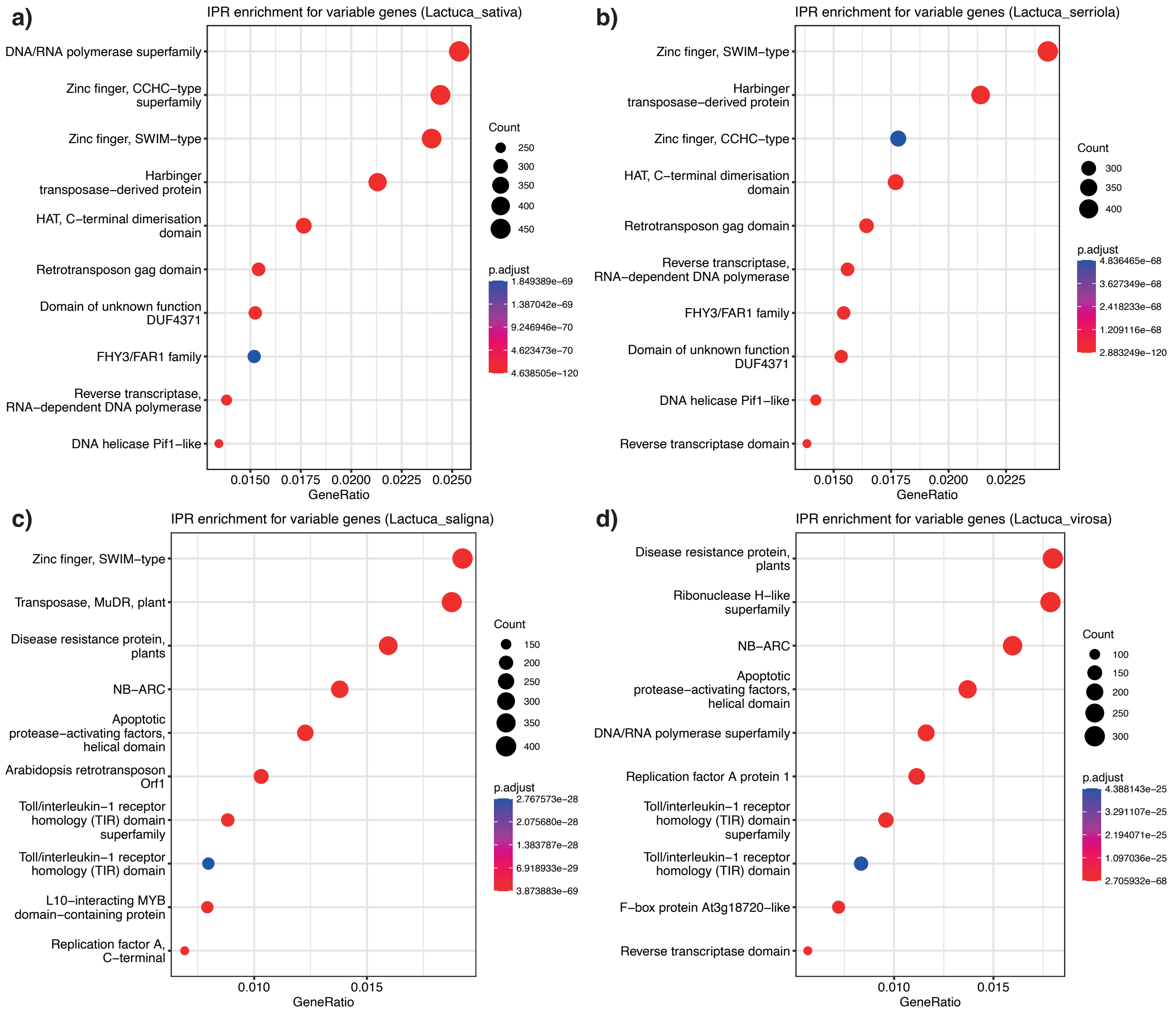

Supplementary Figure S2: Functional enrichment of the variable *Lactuca* genes in *Lactuca sativa* (**a**), *Lactuca serriola* (**b**), *Lactuca saligna* (**c**) and *Lactuca virosa* (**d**) using InterPro domains. Only the first ten most significant domains are shown (p-value adjusted according to Bonferroni). For full results, see Supplementary Data 9.

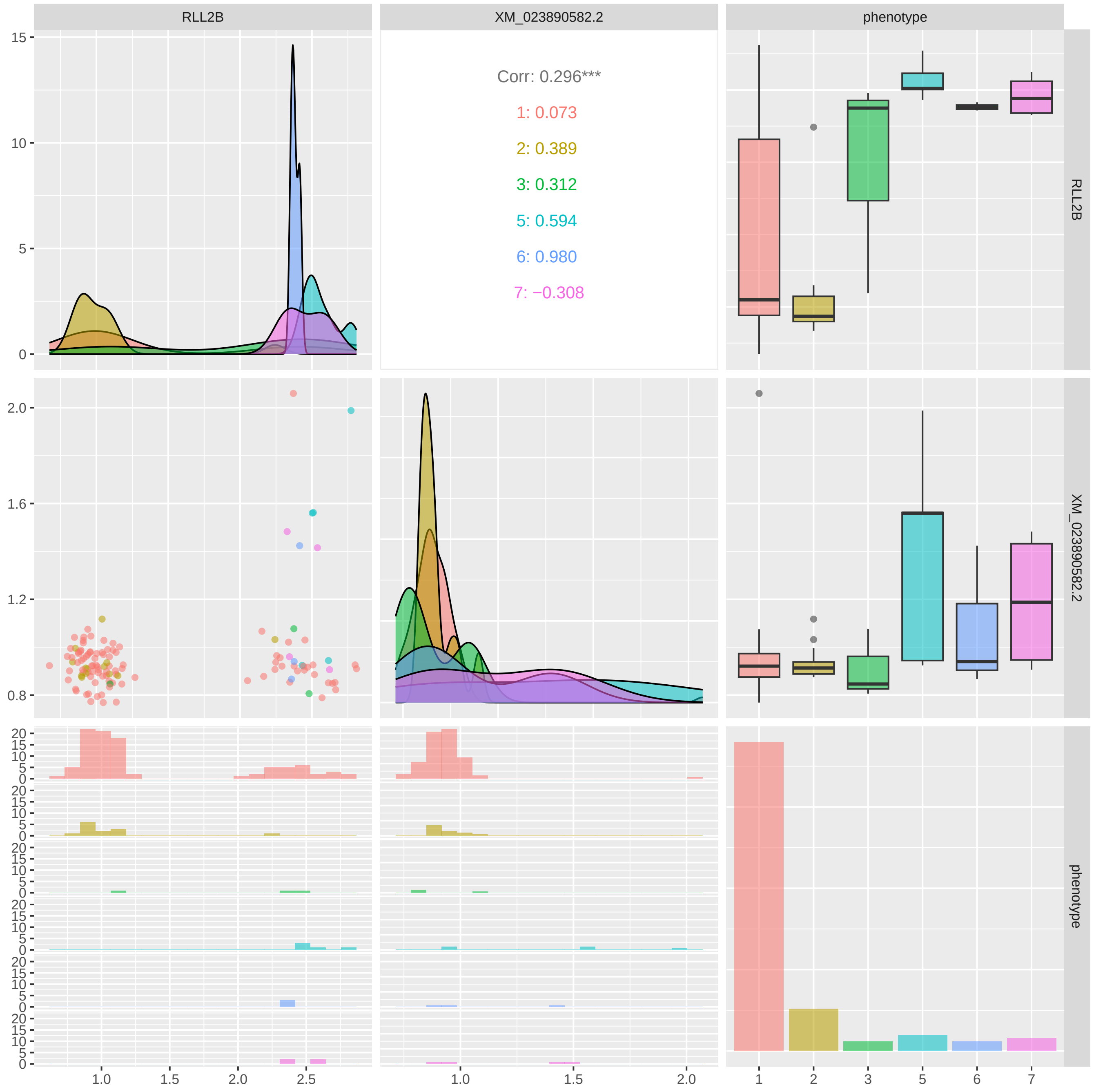

Supplementary Figure S3: Correlation of copy-number variation (CNV) and phenotype values for the "LeafAnthocyaninContent" CNV-GWAS peak on chromosome 5 is shown here. Column/row 1 has the CNV for *RLL2B* (XM_023889304.2), column/row 2 shows CNV for the most significant mRNA hit (on chromosome 5): XM_023890582.2 and column/row 3 shows the "LeafAnthocyaninContent" phenotype from CGN. The diagonal shows the distribution of CNV and phenotype values for their respective columns/rows. Phenotype values are discrete numbers and therefore shows as barplot instead. The bottom triangle shows all correlation plots between CNV values and phenotype. From these it can be seen that the CNV-GWAS hits is correlated to the *RLL2B* gene. This corresponds to the correlation of *RLL2B* CNV values with the anthocyanin phenotype (top right), indicating that a higher copy number of *RLL2B* indeed correlates with a higher leaf anthocyanin content. This plot was created with the 'ggpairs' function from the R package "GGally".

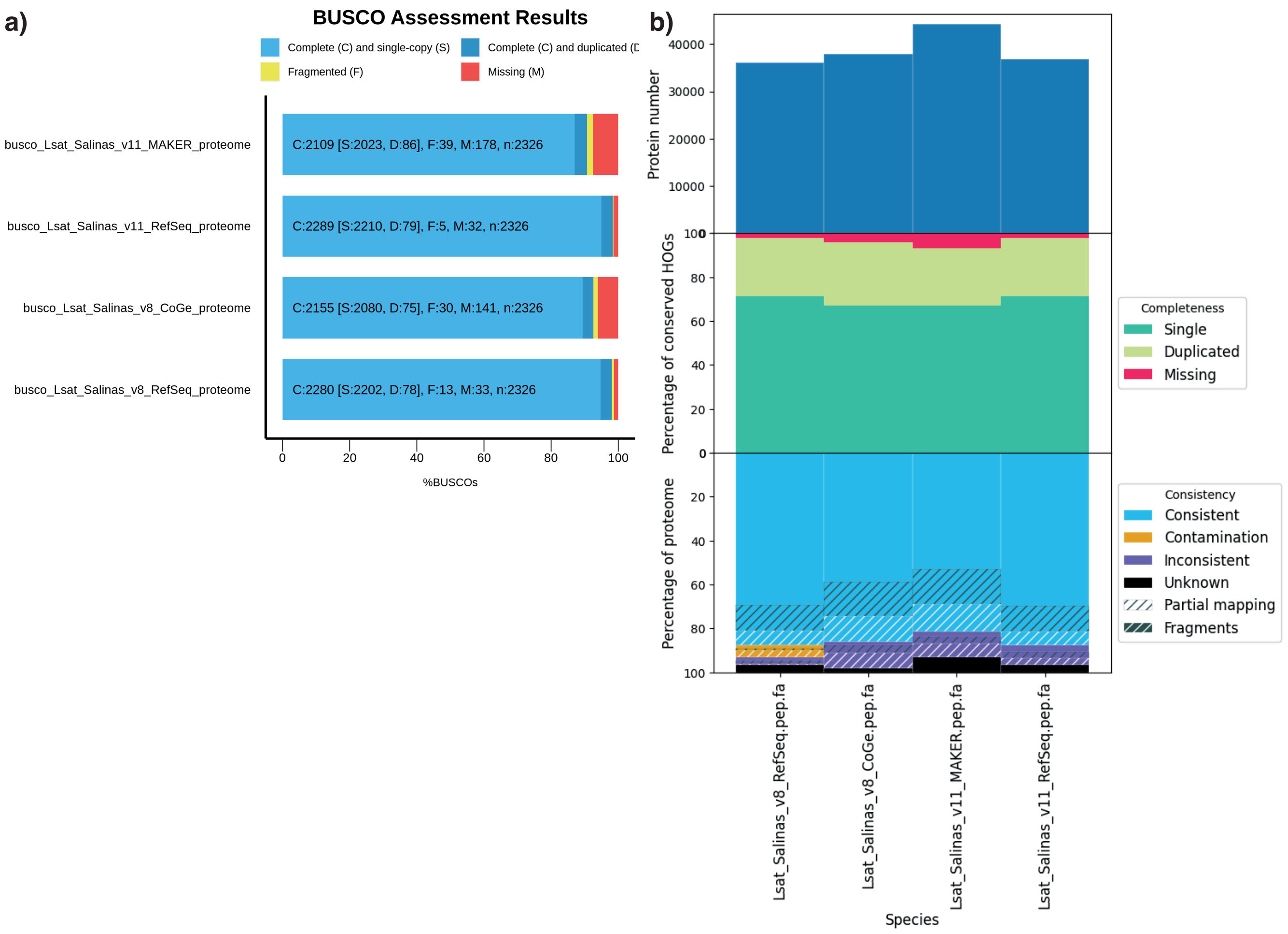

Supplementary Figure S4: Completeness comparison of the proteome for *Lactuca sativa* var. Salinas v8 and v11 with BUSCO v5.2.2 (**a**) and OMArk v0.2.3 (**b**) (Manni et al., 2021; Nevers et al., 2024). For both v8 and v11, the original annotation ("CoGe" and "MAKER", respectively) and the annotation as generated by RefSeq are included.
